# Incidence of gestational diabetes mellitus among Indian women

**DOI:** 10.6026/973206300212763

**Published:** 2025-08-31

**Authors:** Niti Batra, Mohini Ahirwar, Karishma Chaurasia, Manik Sirpurkar

**Affiliations:** 1 Department of Obstetrics and Gynecology, Chhindwara Institute of Medical Sciences, Chhindwara, Madhya Pradesh, India; 2 Department of Obstetrics and Gynecology, Atal Bihari Vajpayee Government Medical College, Vidisha, Madhya Pradesh, India; 3 Department of Obstetrics and Gynecology, Shyam Shah Medical College, Rewa, Madhya Pradesh, India

**Keywords:** Gestational diabetes mellitus, risk factors, incidence, pregnancy, antenatal care

## Abstract

Gestational diabetes mellitus (GDM) is a growing public health concern due to its association with adverse maternal and fetal outcomes, yet its risk factors remain under-investigated in many populations. A prospective observational study of 500 pregnant women who presented on regular antenatal visits to CIMS was completed. During 24-28 weeks of gestation, GDM screening was done through a 75-gram Oral Glucose Tolerance Test (OGTT). Regarding socio-demographic, clinical and lifestyle information, standardized questionnaires were used to collect the relevant data and the risk factors were identified by completing a statistical analysis using SPSS version 25 and multivariate logistic regression. We show a substantial incidence of GDM in women attending CIMS antenatal clinics while, age, obesity; family history and reproductive history remain crucial risk determinants.

## Background:

Gestational diabetes mellitus (GDM) is either the first recognized carbohydrate intolerance in the context of pregnancy or carbohydrate intolerance of varying severity. The incidences of GDM worldwide range from 1-28 per cent of all pregnancies and drastically varyby the population levels and the methods of screening used [1, 2]. The high levels of glucose in maternal bodies expose malnourished mothers and babies to morbidity and mortality, such as macrosomia, hypoglycemia at birth, birth injuries and high chances of contracting type 2 diabetes mellitus in the future [3, 4]. India faces an increasing burden of GDM, with prevalence rates ranging from 7.7% to 21.6%, motivated by the rapidity of urbanization, lifestyle change and heightened cases of obesity among women of reproductive age [5]. Determination of risk groups and knowledge of epidemiology in different regions of GDM is still necessary to achieve proper preventative measures and health policy plans [6]. The risk factors that are traditionally related to GDM are advanced maternal age, obesity, a history of diabetes mellitus in the family, previous GDM, polycystic ovarian syndrome (PCOS), multiparity and prior macrosomic infants [7]. However, variations across geographical regions, ethnicity and socioeconomic status suggest the need for localized studies to inform region-specific interventions [8]. Therefore, it is of interest to bridge this gap by evaluating the incidence of GDM and investigating associated socio-demographic and clinical risk factors among women attending antenatal clinics at CIMS, a tertiary care teaching hospital serving a diverse local population.

## Materials and Methods:

## Study design and setting:

The setting where a prospective observational study took place is at Antenatal Clinic of Chhindwara Institute of Medical Sciences (CIMS), Chhindwara, MP, India and was performed between August 2024 and July 2025, after approval from the Institutional Ethics Committee [Ref. No. CIMS/EC/2024/8513].

## Participants:

Three hundred and fifty pregnant women between the ages of 18-40 who were attending the antenatal clinics were enrolled after giving informed consent. Pre-existing diabetes mellitus, chronic systemic illness, or unwillingness to participate proved to be known exclusion criteria.

## Data collection:

The screen was carried out on all subjects at 24-28 weeks of gestation with a 75-gram Oral Glucose Tolerance Test (OGTT) according to IADPSG criteria. GDM was defined as fasting glucose level of >= 92 mg/dl, 1 hour = >=180 mg/dl and/or 2 hours = >=153 mg /dL. Structured questionnaires (socio-demographic and clinical-related data, such as age, BMI, parity, family history andpast obstetric history) were used to collect data.

## Statistical analysis:

The analysis of data was done in SPSS version 25. A descriptive statistic, Chi-square and logistic regression were carried out. A p-value < 0.05 was taken to be significant.

## Results:

The study enrolled 500 pregnant women, of whom 62 (12.4%) received a Gestational Diabetes Mellitus (GDM) diagnosis. It was found that there were considerable links between certain risk factors and the occurrence of GDM. Women ≥30 years had significantly higher incidence (OR=3.02; 95% CI: 1.61-5.67; p=0.002) (Table 1 - see PDF). Elevated BMI (>25 kg/m^2^) doubled the risk (OR=2.87; 95% CI: 1.54-5.33; p<0.001) (Table 2 - see PDF, Figure 1 - see PDF). Multiparity and family history significantly increased GDM risk, while prior macrosomic birth or stillbirth also correlated significantly (p<0.05) (Table 3 - see PDF). History of abortion, hypothyroidism and Multigravida showed a statistically significant association with GDM (p < 0.05). Sedentary lifestyle, present in 36 of the GDM-positive women, emerged as one of the most significant modifiable risk factors (p = 0.003). In contrast, higher parity (≥2) did not show a significant association with GDM (p = 0.312), suggesting that parity alone may not be a reliable predictor in this population. These findings highlight the multifactorial nature of GDM and underscore the importance of early identification and lifestyle interventions in high-risk groups (Table 4 - see PDF). Figure 2 (see PDF) shows the comparison of risk factor distribution between GDM and non-GDM groups.

## Discussion:

This study highlights a GDM incidence rate of 14.9%, consistent with previous reports from India that range from 7.7% to 21.6% [5]. The findings underscore the necessity of structured universal screening strategies in antenatal settings to enhance early detection and management, ultimately reducing maternal-fetal morbidity and mortality. Advanced maternal age emerged as a substantial risk factor for GDM, aligning with global epidemiological patterns [2, 3]. Increased maternal age potentially exacerbates insulin resistance and beta-cell dysfunction due to chronic low-grade inflammation and progressive decline in insulin sensitivity [1, 4]. Obesity's prominent role in GDM development was confirmed, with overweight or obese women exhibiting significantly elevated risk due to adiposity-driven insulin resistance [6, 8]. Family history of diabetes mellitus is recognized as a genetic and environmental risk factor, indicative of a pre-existing predisposition toward insulin resistance or beta-cell dysfunction, further increasing pregnancy-related metabolic stress [4]. Multiparity and adverse obstetric histories [9, 10-11], including macrosomia and stillbirth, emphasize the cumulative effect of insulin resistance and inadequate glycemic control across pregnancies [7, 8, 12]. Local epidemiological data from this study necessitate tailored interventions at antenatal clinics emphasizing lifestyle modification, dietary counseling and regular monitoring for high-risk groups. Future research should further explore interventions and cost-effective screening strategies tailored to regional needs, addressing socio-cultural and demographic variations in Chhindwara, Madhya Pradesh [13, 14, 15, 16- 17].

## Conclusion:

A significant incidence of GDM among women attending antenatal clinics at CIMS, Chhindwara, underscoring the importance of routine screening in high-risk groups is shown. Maternal age, elevated BMI, family history of diabetes mellitus and multiparity significantly increase GDM risk. Clinicians should focus on preventive strategies, individualized management plans and increased awareness to minimize adverse maternal-fetal outcomes.

## References

[1] Dam N (2025). Diabetes Asia Journal..

[2] Mantri N (2024). BMC Public Health..

[3] Gracelyn LJNS (2016). Int J Reprod Contracept Obstet Gynecol..

[4] Gupta K (2025). Int J Med Res Rev..

[5] Bahl S (2022). BMC Pregnancy and Childbirth..

[6] Gupte S (2023). Int J Diabetes Dev Ctries..

[7] Mustaniemi S (2018). Endocr Connect..

[8] Ye W (2022). BMJ..

[9] Alduayji MM, Selim M. (2023). Cureus..

[10] Dissassa HD (2023). BMJ Open..

[11] Mdoe MB (2021). BMJ Nutr Prev Health..

[12] Larebo YM (2021). Biomed Res Int..

[13] Lagakodie S (2017). Malays Fam Physician..

[14] Muche AA (2019). BMC Pregnancy Childbirth..

[15] Yahaya TO (2020). Egyptian J Med Human Genetics..

[16] Nigatu B (2022). Clin Diabetes Endocrinol..

[17] Meharry MP (2019). Diabetes Res Clin Pract..

